# A Critical Review on the Factors Affecting the Bond Strength of Direct Restorative Material Alternatives to Amalgam

**DOI:** 10.3390/ma17194853

**Published:** 2024-10-01

**Authors:** Zeynep Batu Eken, Nicoleta Ilie

**Affiliations:** Department of Conservative Dentistry and Periodontology, LMU University Hospital, LMU Munich, Goethestr. 70, 80336 Munich, Germany; nicoleta.ilie@med.uni-muenchen.de

**Keywords:** bond strength, glass ionomer cement, resin-modified glass ionomer cement

## Abstract

This article comprehensively reviews the performance of simplified direct restorative materials that have the potential to be an alternative to amalgam. Following an understanding of the material structures and clinical performances, this review provides an analysis of the bonding mechanisms and influential factors on the bond strength. These factors include substrate-related variations, involving primary vs. permanent dentin, sound- vs. caries-affected/demineralized dentin comparisons and surface-related factors and pretreatments. Special attention is given to the factors changing the substrate surface, such as different contaminants, remedy methods after contamination and different conditioning methods related to the materials. Variations in sample preparation and bond strength test parameters are also evaluated for the analysis of the outcomes. This review aims to provide an overview of the factors involved in the application procedure of direct restorations together with in vitro testing variations to guide the selection of suitable materials by understanding strengths and shortcomings.

## 1. Introduction

The long-term success of a restoration depends closely on patient, operator and material factors. Resin-based composites (RBCs) are the first choice of material for direct restorations; however, their use is cost- and time-intensive, and their durability is insufficient under cariogenic environments and challenging clinical situations. Achievement of specific requirements, such as proper isolation and multiple technique-sensitive steps, can be difficult to attain, especially in pediatric and geriatric dentistry. Less technique-sensitive materials suitable for such conditions that bond better to low bonding-receptive tooth structures are needed to develop alternatives for amalgam. Understanding the risk factors can facilitate dentist’s restorative treatment decisions and consequently increase the durability of restorations [1]. The observed current trend is to develop simple-to-use materials with additional therapeutic potential in order to reduce the secondary caries formation and therefore the number of restoration replacements [2]. There are many alternatives available on the market, such as glass ionomer cements (GICs), resin-modified glass ionomer cements (RMGICs) or self-adhesive RBCs with bioactive properties. Therefore, the selection of the most suitable material for specific clinical situations has become another challenge for clinicians. 

GICs have been widely used in dentistry since they were invented by Wilson and Kent in the early 1970s [3]. GICs found on the market vary in their composition, mixing/setting technique, powder/liquid ratio and viscosity, which consequently influences their indications. While light curing is needed to initiate the radical polymerization of RBC, the setting reaction of GICs is an acid–base reaction initiated by mixing calcium fluoro-alumino-silicate glass containing powder and aqueous poly(acrylic acid) (PAA) solution [4,5]. 

GICs have the advantages of being biocompatible with the pulp, being capable of releasing fluoride, allowing bulk application, requiring short application times and chemically adhering to enamel and dentin without necessitating an intermediate agent [6]. Despite their beneficial properties, GICs are brittle and susceptible to water uptake and loss during the initial setting reaction, which can lead to deterioration in mechanical properties [5]. The initial hardening of the material takes place in a short time (2–6 min) following mixing. The cement continues to undergo changes in the following days or even months, which is known as maturation [7,8]. The freshly set cement is susceptible to water exchange. During setting, the cement should be protected from additional water to prevent loss of metal ions. In addition, the loss of water can cause the cement to dry out, resulting in the formation of micro-cracks and a chalky appearance [9]. Their insufficient mechanical properties limit their clinical indications to the restoration of primary teeth, cervical lesions, temporary restorations, the intermediate base in the sandwich technique and atraumatic restorative treatment (ART). 

To overcome the shortcomings of conventional GICs (CGICs), variations of these materials have been developed [10]. The high-viscosity GICs (HVGICs) contain smaller fluoro-alumino-silicate glass fillers, more fluorite and an increased powder/liquid ratio in order to achieve rapid setting and higher strength. By optimizing the polyacid and particle size distribution, these materials were marketed as suitable for posterior stress-bearing restorations [10,11]. In the last few years, efforts have been made to develop nano-filled polymer coatings to seal and interfere with the surface of the material aiming to improve the flexural strength [12]. Some of these systems are advertised as “glass hybrid” materials, which is inappropriate terminology and will be avoided in this review. 

Other widely used materials are the RMGICs, which were marketed as hybrid materials combining the advantages of GICs and RBCs. Methacrylate-based monomers (e.g., hydroxyethyl methacrylate (HEMA)) and photo-initiators are included in the composition. These materials retain the acid–base reaction of the GICs and include a polymerization reaction of methacrylate monomers [13]. Addition of resin in the composition provided the advantages of reduced setting time, lower early-moisture sensitivity and improved mechanical properties [14,15]. The acid–base reaction starts by mixing the powder and liquid, and the polymerization reaction of the monomers occurs by light activation. These two reactions compete with each other and impact each other [16]. Due to the presence of monomers, their biocompatibility is less than that of GICs [17]. RMGICs exhibit high water sorption and solubility that was attributed to the presence of strongly hydrophilic HEMA. In particular, lining materials with a reduced powder to liquid ratio result in a potential of higher water uptake [18].

The high desire to further simplify the clinical application of restorative procedures led to the development of novel self-adhesive materials. Manufacturers introduced new materials to the market that have the ability to release caries-protective ions, using the term “bioactive” [19,20]. Although these materials are marketed as a new material category, they can be viewed as either the chemical advancement of known material categories such as self-adhesive RBCs, compomers, giomers, GICs and RMGICs or as a complex hybridization of these material categories [20,21]. One of these materials is similar to an RBC with additional fillers with ion-releasing properties, while others can be considered modifications of RMGICs due to their composition and acid–base reaction. These materials have been shown to be promising regarding biological [22] and bioactive properties [23,24].

The listed materials have different technical sensitivities and aging behavior. In addition, they adhere to the tooth structure via different mechanisms and can be influenced to varying degrees by patient- and environmental-specific parameters. Since bond strength is considered a valuable parameter for assessing the performance of a new material in advance of clinical use, the aim of the study was to analyze and critically review the parameters that can significantly affect bond strength in order to guide selection for materials suitable to be an alternative to amalgam. In a preamble to factors related to bond strength, the clinical behavior and bonding mechanisms of the restorative materials are summarized. 

## 2. Materials and Methods

The authors identified the most common clinically relevant factors that have potential influence on the bonding performance of GICs. The literature search was carried out using PubMed with the combination of the following keywords: glass ionomer, resin-modified glass ionomer, bond strength, primary/deciduous, dentin, contamination, saliva, blood, hemostatic agent, pretreatment, conditioner. In addition to the articles identified using these keywords, relevant articles quoted in the reference list of the retrieved studies were also included. Studies evaluating the clinical performance and in vitro bond strength of GICs were included. The studies evaluating the GICs as adhesives, luting agents or sealants, as well as the studies using methodologies other than bond strength tests, were excluded. 

## 3. Results and Discussion

### 3.1. Clinical Performance of the Material Alternatives to Amalgam

Clinical trials evaluating and comparing different material categories allowed the researchers to observe the long-term functional and esthetic behavior of the materials in a complex oral environment. In addition to the material-related factors, longevity of restorations is influenced by patient- and tooth-related factors. These factors include caries risk, parafunctional habits, age, number of restored surfaces and practitioner’s experience [1,25,26,27,28]. The valuable data obtained by the clinical trials guide the development and improvement of the restorative materials. 

#### 3.1.1. Clinical Performance of RBCs

RBCs have high chance of survival in load-bearing regions [29]. Long-lasting survival of RBCs in posterior regions has been proven by an abundant number of clinical trials [25,26,30,31,32,33,34]. The potential of RBCs to perform for decades was shown after 27 years [30], 30 years [31] and 33 years [32]. The latter retrospective practice-based study reported an annual failure rate of 2.4% after up to 33 years of function [32], similar to the other clinical trials [33,35]. The survival rate of Class I and II restorations restored with RBCs was found to be higher than that of GIC restorations (HVGIC and RMGIC together) [36]. However, Peumans et al. [37] reported a lower annual failure rate for GICs compared to RBCs for non-carious cervical lesions in terms of retention. The clinical performance of RBCs is influenced by the type of adhesive system. Simplified adhesive systems (one-step self-etching) perform worse than multistep adhesive systems (two-step self-etching and three-step etch-and-rinse) and GIC-based materials when restoring cervical lesions [38,39]. 

The main reasons for failure of posterior RBC restorations are caries at the restorative margins and material fractures [28,36]. The patient caries risk factor plays an important role in the survival of the restoration. Medium and high caries risk increases the risk of restoration failure by two to three times [28]. Secondary caries develop at the biofilm stagnation areas, where the plaque cannot be removed easily by saliva, mastication and brushing. They are located gingivally more frequently than in the occlusal margin, irrespective of the material selection [40]. Replacement of the restoration is the most often-applied treatment in the case of secondary caries, and this adds a heavy burden on the health care system [41], accounting for more than half of restorations applied by practitioners [42].

#### 3.1.2. Clinical Performance of GICs

GICs can be the material of choice for their fluoride-releasing and remineralizing capability [43]. The clinical studies testing the performance of HVGICs as a permanent restorative material showed sufficient durability and good clinical performance when compared with RBCs for Class I and II restorations, even at a 10-year follow up [44,45,46]. However, increased wear was observed with HVGICs [46]. It was also shown that after 3–4 years, there is a moderate to high risk of failure, with a high number of fractures in Class II restorations with HVGICs, likely related to lower-strength properties [47,48]. This limits their indications with small or midsize Class II cavities in permanent teeth [10,29,36,48]. A recent umbrella review reported higher failure rates of HVGICs than RMGICs for the restoration of carious primary teeth at 36 months [49]. In Class II restorations of primary teeth, GICs and RBCs present similar clinical performance, except for the superior performance of GICs regarding the occurrence of secondary carious lesions, especially with RMGICs under rubber dam isolation [50].

GICs also have broad indications in Special Care Dentistry. In the case of a disability, ART or operative treatments under general anesthesia are the alternatives for the conventional treatments. ART is a proven approach, wherein a GIC is used for the restoration following the manual excavation of the caries [51]. A 5-year follow-up clinical trial compared the survival of ART restorations using HVGICs and conventional RBC restorations in the clinic or with general anesthesia, used for patients with disabilities. HVGICs using ART showed higher longevity than RBCs overall. The lower survival percentages for RBCs placed in-office compared to general anesthesia reflect the difficulty of moisture control under challenging situations in the office [52].

#### 3.1.3. Clinical Performance of RMGIC

In cervical lesions, RMGICs had a higher chance of survival and retention rate compared to RBCs [29,53]. Long-term (13 years) dentin retention of RMGIC to non-carious cervical lesions has been found to be the highest, together with four-step etch-and-rinse adhesive, compared to other self-etch and etch-and-rinse adhesives [54]. Despite their good retention, the poor esthetic properties of GICs may limit their indications, especially in the anterior region [38,55].

For the restoration of Class II cavities in primary teeth, RMGIC has been found to be more successful than GIC [56,57]. However, a similar overall success rate was found compared to RBC after 2 years, except for higher occlusal wear of RMGIC [58]. Recently, a flowable RMGIC that was initially marketed with a short phosphoric acid treatment recommendation was compared with an RBC for the restoration of Class I and II cavities after a one-year follow-up. This RMGIC was applied without the use of an adhesive, which resulted in an unacceptably high failure frequency. It was recommended that further studies should be performed using a bonding agent [59]. Thereafter, the manufacturer included the recommendation of using the material together with an adhesive system. When applied with an adhesive system, the material showed functionally and esthetically similar clinical performance to bulk-fill RBC after one year [60] and to a compomer after a two-year follow-up [61]. 

Longitudinal clinical trials are the ultimate way to assess the effectiveness of a restorative material; however, they are expensive and time-consuming. Although the clinical performance of GICs and RMGICs is promising, the in vitro results do not necessarily indicate a similar direction with clinical trials [62]. With the rapid evolution of restorative materials, the popularity of in vitro studies to predict clinical effectiveness has increased.

### 3.2. Bonding Mechanisms

It is crucial to understand the bonding mechanism of the materials in order to analyze their performances in different situations and to understand and correct the errors during the treatment. Basically, the bonding mechanism of RBCs, along with adhesive systems, involves the flow of resin monomers into demineralized tooth substrate, leading to micromechanical interlocking and hybridization. Additionally, chemical interaction is achieved by specific monomers in an self-etching approach [2]. GICs and RMGICs are self-adhesive materials that bond to dentin chemically and micromechanically. These materials are hydrophilic in nature, providing the capability of wetting the tooth structure [63]. 

The adhesion of GICs initially involves a chemical bond, occurring by the formation of ionic bonds between the calcium ions in the hydroxyapatite and the free carboxyl groups of the polyalkenoic acid [64]. Micromechanical interlocking forms by the infiltration of the material into the exposed collagen network at the surface. The diffusion process of ions from the cement and the tooth creates a slowly formed interfacial zone, the ion exchange layer or acid–base resistant layer [65,66,67,68].

### 3.3. Factors Affecting the Bond Strength 

**Figure 1:**
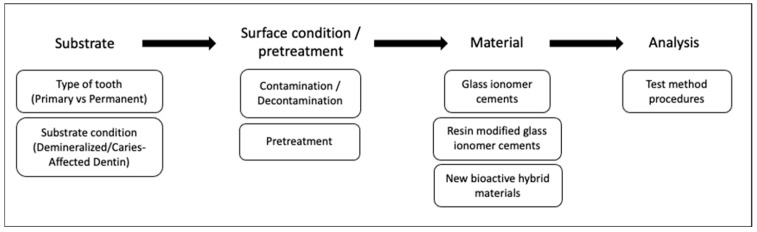
Evaluated factors affecting the bond strength of direct restorative material alternatives to amalgam.

During the application of a restorative material, clinicians face many challenges or situations that need specific approaches. These include (1) substrate-related factors differing among patients, such as primary or permanent teeth and sound- or caries-affected dentin, (2) factors affecting the substrate surface, such as contamination or pretreatment prior to restorative material or (3) factors related to material properties. These situations potentially affect the interaction between tooth and material, affecting long-term survival. Assessment of these complex factors is not always applicable in clinical trials but is possible with standardized bond strength tests. In addition, differences between testing and analysis methods may affect bond strength results. Therefore, influence of these clinically relevant factors and some testing variables on the bond strength of GICs will be discussed below (Figure 1). 

#### 3.3.1. Type of Tooth: Primary vs. Permanent Teeth

Considering the patient-related factors, such as cooperation and caries risk in pediatric dentistry, the choice of material plays an important role [10]. Clinical and structural differences between primary and permanent teeth make it necessary to evaluate the bonding characteristics of restorative materials on both substrates [69]. Primary teeth present structural differences such as reduced thickness of dentin, larger pulp chamber [70] and higher tubule density [71] with large tubule diameters compared to permanent teeth [69]. In order to obtain a sufficient surface area in dentin to bond in laboratory test, the distance of the prepared dentin in primary teeth is likely to be closer to the pulp compared to the permanent teeth due to the lower thickness of primary dentin. This would likely expose wider dentin tubules. The primary teeth used in laboratory studies are generally exfoliated; therefore, the pulp chamber is in contact with the environment, which would affect the water content [72]. Structural differences in deep dentin and increased water content may lead to reduced bond strength [73]. 

Primary dentin has been found more reactive to conditioners, causing deeper demineralization of the intertubular dentin and a thicker hybrid layer [74]. Excessively deep demineralization causes insufficient primer and adhesive resin infiltration. Non-impregnated demineralized dentin at the bottom of the hybrid layer is the weakest part, which may act as a pathway for microleakage and make it susceptible to failure and reduced bond strength [75]. Therefore, shorter conditioning with adhesive restorations for dentin was suggested for primary dentin [74,76]. 

A systematic review and meta-analysis evaluating in vitro studies showed that the bond strength of adhesively placed RBCs to permanent dentin was higher than to primary dentin [77]. Although GICs have been commonly used in pediatric dentistry for a long while, information on the comparison of GIC bond strength to primary and permanent teeth is limited [72,78,79,80,81,82]. Burrow et al. [72] reported that the overall bond strengths of CGIC and RMGIC as material categories on the permanent dentin were greater than to primary dentin, confirming an earlier study [79]. However, in the same study, none of the materials individually showed significant differences between the dentin types [72], which is in line with several other studies [80,81,82]. 

In healthy primary dentin, RBCs bond better than RMGICs [82,83,84,85,86,87,88,89], with a few exceptions where both bond similarly [90]. Similar to the permanent dentin [91,92,93,94,95], superior adhesion of RMGICs to sound primary dentin was shown compared to GICs [83,88,96,97,98,99]. Recently, a new RMGIC combined with a bonding agent was evaluated in two studies and showed higher bond strength than RMGIC [100] and CGIC [101], which can be easily related to the bonding agent.

The majority of the studies evaluated the bonding capacity of GICs to primary dentin following a very short 24 h storage. However, GIC maturation takes place over time, improving the physical properties of the material [7]. On the other hand, stronger gelatinolytic activity after acid-etching was reported in primary dentin compared to permanent dentin, possibly related to the structural differences [102]. Therefore, long-term evaluation of GICs bonded to primary dentin should be a topic of interest. HVGIC results were stable after one year [83] but reduced after two years of water storage compared to bond strength results after 24 h [97]. Although RMGIC bond strength was not influenced by one year of water storage [83], nor by simulated aging through mechanical stress and cariogenic challenge [85], the results after two years of water storage were material-dependent [97]. There appears to be an effect of substrate pretreatment on the degradation of RMGICs. When comparing the bond strength of two RMGICs applied with PAA or HEMA containing light-curing primer, degradation of the RMGIC with the primer was reported on both primary and permanent dentin following immersion in 10% aqueous solution of sodium hypochlorite. The authors related this result to the solubility of primer with high HEMA content [78]. 

#### 3.3.2. Tooth Condition: Demineralized/Caries-Affected Dentin vs. Healthy Dentin

**Figure 2:**
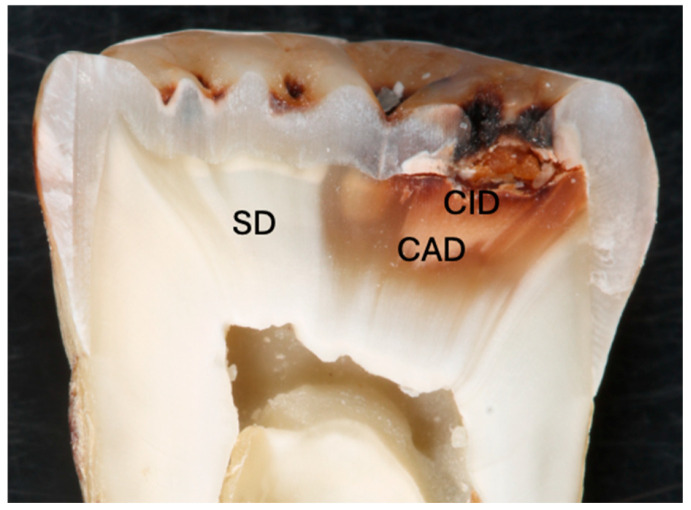
Cross section of a molar tooth with occlusal caries lesion showing caries-affected dentin (CAD), caries-infected dentin (CID) and sound dentin (SD).

In vitro studies most often involve bonding to sound dentin; however, there are a variety of dentin structures in clinical practice, including reparative, sclerotic, carious, demineralized and hypermineralized. These different structures may have influence on the long-term adhesion of the restorative materials [103]. With a better understanding of the pathophysiology of the caries process and the remineralization potential of the materials, selective caries removal gained more interest. Caries lesion consists of an outer caries-infected dentin (CID) layer and a deeper caries-affected dentin (CAD) layer (Figure 2). According to minimal invasive dentistry, CAD and sometimes CID (in case there is the risk of pulp exposure) should be preserved following the removal of the outer soft substantially demineralized CID [104,105,106,107]. Therefore, restorative materials are bonded to the CAD in the cavity floor, which is different to sound dentin morphologically, chemically and physically, as well as to sound tissue in the peripheral enamel and dentin. Furthermore, complete removal of carious tissue is not possible in the ART, where caries removal is completed with hand instruments [108].

CAD exhibits lower mineral content [109], a higher degree of porosity, greater water content [110] and altered collagen organization [111]. Reduced hardness, reduced nanomechanical properties and lower cohesive strength of CAD were shown compared to sound dentin [112,113]. As a response to the caries process, dentinal tubules are occluded by the apatite crystals (intratubular deposition of calcium phosphate crystals) and tubule diameter decreases to prevent bacteria and toxic materials from permeating [114]. Moreover, large rhombohedral crystals of Mg-substituted β-tricalcium phosphate crystals (whitlockite) are deposited within the sclerosed dentinal tubules. This occurs by depletion of Mg ions from the medium or partial transformation of β-tricalcium phosphate crystals to apatite [115].

Laboratory models have been developed to simulate the artificial caries lesions in enamel and dentin in order to overcome the difficulties of working with natural caries lesions (Table 1). Artificial models make it possible to standardize and predict the characteristics of the lesions, such as depth and mineral loss [116]. These models are commonly used in in vitro studies for the analysis of remineralization, the preventive effect of the restorative materials or the bonding capacity of the materials to the demineralized tooth structures [93,117,118]. However, the natural caries process is more complex than the artificial caries lesion formation. It consists of dynamic, continuous phases of demineralization and remineralization [104]. Artificial caries-affected dentin (ACAD) simulation models are an alternative for simulating natural caries-affected dentin (NCAD) in order to eliminate the substrate variability and achieve standardization with uniform and flat surfaces in the laboratory. These models include static (acidified gels) [119,120], dynamic (pH cycling) [117] and microbiological (bacteria) [121] methods. Further, “pH cycling” refers to more complex protocols that involve cycles of demineralization and remineralization, with mineral uptake and loss and pH similar to the natural process [117,122]. Marquezan et al. [123] compared different artificial caries methods on primary dentin in terms of hardness and morphology and reported that pH cycling is a more appropriate method for the simulation of CAD obtained after caries removal, while the microbiological method simulates the caries lesion with the infected layer prior to caries removal. Although it has been shown that ACAD can simulate NCAD [123,124], ACAD that is created in a shorter period of time results in fewer crystals occluding the dentinal tubules [124]. In vitro models have the capability of simulating chemical changes, creating the demineralized surface, but the dentin–pulp complex response of tubular occlusion is not sufficient [85]. Therefore, the results of the studies with different methods of artificial caries preparation methods or NCAD should not be compared. 

It is known that the bonding of adhesive systems to CAD is compromised compared to sound dentin [125,126,127]. Lower hardness, a thicker hybrid layer and poor resin infiltration were recorded along with reduced durability compared to sound dentin [128]. The higher amount of water in the exposed organic matrix in the demineralized dentin is also possibly related to low bond strength. The water would impede the penetration and polymerization of the restorative material [109]. 

Due to the remineralization potential of CAD and GICs’ resistance to moisture, evaluation of GICs on this demineralized substrate is important. Since the bonding mechanism of GICs depends on chemical bonding between calcium from hydroxyapatite and carboxylic groups of the polyacrylic acid [64], reduction in the bond strength of GIC to demineralized dentin would be expected due to the lower amount of minerals. CAD is not rich in minerals and additional use of cavity conditioner removes the remaining minerals even further, thus reducing the bonding ability and causing the bond strength to depend on the infiltration around the exposed collagen fibers [94]. 

The main features of the studies comparing the bond strength to sound and CAD are shown in Table 2. Most of the studies that compared the bond strength of GICs and RMGICs to sound and NCAD failed to show reduction with altered substrate [94,129,130,131,132]. On the other hand, several studies compared the performance of GICs and RMGICs on sound and ACAD prepared with different methods. When the demineralization was created by the static method [87,93,133,134] and microbiological method [135], GICs showed lower bond strength compared to sound dentin. The studies using the pH cycling model exhibited controversial results. Specifically, pH cycling mostly resulted in insignificant differences compared to sound dentin [85,97,98,136], while reduced results were also shown in few studies [91,137]. The studies that reported no difference with pH cycling were fulfilled on primary dentin, while differences were shown on permanent dentin. 

Among the studies that compared the performance of different restorative materials on demineralized dentin, RMGIC performed better than GIC [83,91,93,97,98,144,145], in contrast to a few studies showing similar results [132,142]. Although the bond strength of RBCs is generally affected by the demineralized surface [83,85], they showed higher bond strength than RMGIC and GIC [83,131,132,134,142]. The influence of erosive challenge that also caused reduction in mineral amount on the dentin surface was evaluated in two studies [83,142]. Even though HVGIC was not affected by the erosion, the same RMGIC tested in the studies showed different results. The wide variety of methodological differences among these studies, such as demineralization method, tooth type (permanent/primary), conditioner or material selection, should be taken into consideration while evaluating the studies. Therefore, the results cannot be directly compared. 

Demineralization of the dentin substrate makes the adhesive interfaces more prone to hydrolytic degradation than sound dentin [128]. Among the studies evaluating durability of the materials bonded to CAD, reduction in the bond strength of RMGIC was observed following 3 months [131,133] and 1 year [146]. However, three other studies evaluating the same RMGIC brand applied with its own primer containing HEMA found it to be durable after simulated aging [85] for 1 year [83] and 2 years [97]. Pretreatment of the already poorly mineralized dentin with primer rather than PAA conditioning could be a possible reason for the durability. HVGICs were also found to be stable over time [83,131]. Ions released from GICs have been shown to penetrate into the demineralized dentin [147,148] and protect the degradation of exposed collagen [106]. However, mineral uptake is not enough to conclude GIC remineralization of apatite-depleted collagen matrix along the lesion surface [148]. It would also be an extrapolation to make a suggestion over the remineralization potential of GICs from the bonding strength results on demineralized dentin over time. 

#### 3.3.3. Surface Condition: Contamination

Rubber dam isolation is recommended in an ideal restorative treatment for the control of moisture and prevention of the contamination of the field with saliva and other fluids. It was shown that the use of the rubber dam increased the longevity of restorations [149]. However, in clinical situations such as cervical lesions, cavities at the gingival margin, malpositioned teeth, incomplete eruption of the tooth or uncooperative patients, especially in pediatric and geriatric dentistry, it is difficult to place a rubber dam to obtain complete dryness. The cervical region is the area where the rubber dam clamp is placed, and therefore, it is difficult to isolate from the cervical fluid. In such situations, GICs are a good alternative, with fewer and simpler steps to diminish the event of error during the multiple technique-sensitive steps, as well as reducing the treatment time. 

In clinical practice, there are several contaminants that would affect the adhesion of the restorative material to the substrate. Water, organic debris and/or biofilms hinder the interaction of the materials [150]. Saliva is the most common contaminant, being omnipresent in the oral cavity and composed mostly of water and macromolecules like proteins, glycoprotein sugars, amylase and inorganic particles [151]. Saliva has been shown to negatively influence the bond strength of dentin bonding agents [152]. Saliva-contaminated surfaces prevent interaction with the material and the complete infiltration of the resin. Drying the saliva leads to evaporation of the water, leaving a film of glycoproteins on the surface [151], and the dentin tubules can adsorb these glycoproteins [153].

Table 3 provides the relevant information and main findings of the studies evaluating the effect of different contaminants on the bond strength of GICs and RMGICs. A limited number of studies evaluating the effect of saliva contamination on the dentin bond strength of GICs and RMGICs reported controversial results [154,155,156,157,158,159]. A few of these studies showed a detrimental effect of saliva on GIC and RMGIC bonding performance [154,159,160]. In two of these studies, saliva was applied for a long period of time (1 min, 2 min or 10 days), and reconditioning with conditioner was found to be effective for recovering the bond strength [154,160]. The saliva exposure time would be effective with regard to these results, since GICs would displace or bond when the adsorbed salivary layer on the surface was thin due to being applied in a shorter time [158]. However, Safar et al. [159] reported that rinsing, drying or reconditioning with 10% PAA failed to result in a bond strength of RMGIC comparable to that for noncontaminated dentin [159]. The authors speculated that the saliva precluded the wetting of the dentin surface by RMGIC, and salivary contaminant was resistant to tested decontamination methods. On the contrary, several studies showed no significant effect of saliva on bond strength [155,156,157,158]. Bond strength of RMGIC was found to be resistant to water and saliva contamination when applied in combination with a self-etching bonding agent, either before or after contamination [157]. When a different group of materials was compared, RBC exhibited reduced bond strength and greater microleakage after saliva contamination, while RMGICs’ and GICs’ bonding performances were not affected [155,156]. Therefore, it was suggested that GICs and RMGICs are more suitable for inhibiting potential secondary caries that result due to microleakage and reduced bond strength when rubber dam isolation is not possible, like with Class V cavities. However, it should be noted that artificial saliva lacking organic components was used in these studies, which might not completely reflect the clinical situation, creating misleading results. 

Gingival tissue in the cervical area and deep Class II cavities, soft tissues or pulp may be sources for the blood in the oral cavity. Blood contamination mixed with saliva and/or gingival fluid commonly occurs in clinical practice [163]. There is a lack of studies showing the effect of blood on the bond strength of GICs, except for one recent study reporting that reconditioning was not able to reestablish the reduced bond strength values following blood contamination [154]. Hemostatic agents are commonly used to achieve bleeding control prior to the placement of a restoration, especially in the cervical area, where it is difficult to control blood contamination. These agents have a detrimental effect on the bond strength of RBCs and resin cements on dentin [164,165]. However, their effect on the GICs has not been evaluated comprehensively. Saad et al. [161] evaluated the effect of hemostatic agents and different conditioning methods on the bond strength of RMGIC to dentin. Hemostatic agents were found not detrimental to the bond strength. However, it should be noted that hemostatic agents were applied prior to conditioning in this study. Although another study [154] showed reduction in the bond strength of RMGIC to dentin following contamination with a hemostatic agent, reconditioning of the surface was found effective to reestablish the bond strength values. Therefore, despite the limited number of studies and the varying types of hemostatic agents available, application of conditioner can be recommended for the removal of the hemostatic agent in case of contamination of the dentin surface. However, reapplication of 10% PAA prior to RMGIC application was found to be ineffective when dentin was contaminated with eugenol-based provisional cement and handpiece lubricant. In this study, cleaning with chlorhexidine and pumice were also found to reduce the bond strength [162].

The limited number of studies and high variability of factors, such as the tested contaminant, quantity, duration, stage of contamination, decontamination method, type or usage of conditioner, material or testing method, makes it hard to compare the results of the evaluated studies. Therefore, comparative analysis of the materials is not suitable. There is still a need for studies focusing on successful decontamination methods, as well as the development of materials tolerant to contaminants. 

#### 3.3.4. Surface Pretreatment 

Removal of the smear layer and partial demineralization of the upper layer dentin create a favorable surface for GIC bonding. Several different agents have been evaluated over the years for the pretreatment of the dental surfaces prior to GIC application. PAA, phosphoric, citric, tannic and ethylenediaminetetraacetic (EDTA) acids were evaluated for their potential to enhance the bond strength. PAA has been a well-accepted conditioner recommended by the manufacturers [166]. It is undesirable to over-etch the dentin with strong acids such as phosphoric acid prior to GIC application [167]. PAA, which is a weak acid, leaves the smear plugs intact, containing calcium and phosphate ions necessary for the chemical adhesion of GICs [65,168]. It is crucial to use the conditioner on bur-cut dentin, although unnecessary on smear-free fractures [169]. The improving effect of PAA is achieved by its cleaning effect, micromechanical interlocking due to partial demineralization and chemical interaction [66]. It was shown that a layer of PAA remains on the surface due to its incomplete removal despite being rinsed off. This thin layer (up to 0.5 μm) has been referred to as the gel phase [66]. Ion exchange between the GIC and the partially demineralized collagen fibrils was speculated to be the result of the formation of this intermediate layer within the smear-depleted dentin [65].

However, the necessity of using PAA conditioner is still not completely clear for both GICs and RMGICs. There are many variables, such as concentration of the conditioner, application time, method of application and the structure and thickness of dentin. Different percentages (10%, 20%, 25%) of PAA were evaluated along with different application times. A systematic review and meta-analysis evaluated the effect of conditioning on GICs and HVGICs, including adhesion, microleakage and clinical studies [170]. It was concluded that conditioning with PAA has a positive effect on the bond strength of GICs; however, the clinical trials failed to confirm this. A group of successive studies have evaluated the durability of a CGIC bonded to dentin with and without PAA pretreatment in different time intervals of 1 week, 1 month, 3 months, 6 months [171], 1 year [172] and 3 years [173]. Although the benefit of conditioning was shown at 6 months, no significant difference was found between conditioned and non-conditioned surfaces after 1 year and 3 years. A remineralized dentin layer on the interface was observed in both tested surfaces after 3 years of in vitro aging [173]. Similarly, needle-like crystals within the remineralized layer and along the collagen fibrils were observed following 1 year of functioning in vivo [174]. Even though several studies failed to show improved bond strength with the application PAA [90,160], it can be accepted that it is beneficial to conditioning the dentin [167,175,176,177]. Nevertheless, Sauro et al. [178] reported a significant bond strength reduction after 6 months of artificial saliva storage of PAA-applied RMGIC, unlike non-conditioned specimens. It was speculated that PAA may increase the hydrolytic degradation at the interface. This result contradicts another study [133], which showed no significant reduction following 3 months of aging. 

Apart from the traditional conditioning methods, lasers have gained interest for dentin pretreatment. However, understanding of their influence on bond strength of GICs and RMGICs is still scarce. An Er:YAG laser for pretreatment showed adverse effects on bond strength compared to conditioning [179,180] and similar effects to no pretreatment [181,182]. However, an Er,Cr:YSGG laser was not found to be detrimental when compared to PAA-treated or non-treated dentin [183,184,185]. Chlorhexidine (CHX) has been revealed to be a matrix metalloproteinase inhibitor that prevents the degradation of etched dentin surfaces [186]. It has also been used as a cavity disinfectant, and it was found that it did not interfere with the adhesion of GIC, RMGIC and bioactive restorative materials [132,187,188]. However, CHX-treated dentin following PAA conditioning exhibited lower bond strength to RMGIC at 6 and 12 months. It was speculated that this might be due to interference of CHX with the bonding mechanism and maturation of the material [189]. Other than PAA, bond strength to EDTA-treated dentin was also evaluated. Although it was found to be similar or better than PAA after 24 h [133,180,190], controversial results were shown after 3 months of aging [133,190]. 

It was also proposed to enhance the bond strength of RMGIC with a dentin bonding agent, since it contains resin. Self-etch or etch-and-rinse adhesive systems, as well as self-etch primer, improved the bond strength of RMGICs [161,175,191]. Self-adhesive restorative materials that have bioactive potentials were also investigated, combined with a bonding agent, and they showed increased bond strength values [192,193]. However, the ion-releasing property of these materials may decrease when used combined with a bonding agent. Even though the adhesive layer may not completely prevent fluoride release, the amount decreases [194,195]. 

#### 3.3.5. Test Method Variables 

Among the discussed factors related to clinical application, there are many variables in laboratory testing that influence the bond strength [196,197]. Substrate-related variables such as type of teeth (human/bovine, third molar/incisor), age of the teeth, depth of dentin (deep/superficial), location of dentin (occlusal/proximal/buccal), surface preparation (bur/abrasive paper), simulation of pulpal fluid flow and surface type (cavity/flat), as well as testing method-related variables such as aging method, specimen storage medium, temperature and time, bond strength test used and testing procedures, have specific impacts on the results [196].

Specimen preparation methods differ between studies, while SiC papers are the most commonly preferred method for flat dentin preparation [198]. The thickness and roughness of the smear layer is affected by the preparation method and has an influence on the bond strength [199,200]. No difference was found between the dentin surfaces prepared with different burs, including carbide, diamond and polymer [201,202]; however, the influence of cavity preparation methods should be further evaluated for GICs. 

Moreover, different methods of caries removal, such as the conventional method, chemomechanical methods and lasers, cause residual dentin of different natures [107]. The concept of chemomechanical caries removal was considered a conservative method in terms of minimally invasive dentistry. When compared with the conventional bur-cut caries removal technique, it appeared to have no adverse effect on the bond strength of GIC [94,203,204], and RMGIC [130,201,205,206]. For selective removal of caries with lower pain and discomfort to the patient, Er:YAG lasers can be an alternative for cavity preparation. Nevertheless, RMGICs bond less effectively to laser-irradiated dentin than to bur-cut dentin [169,207]. However, it was shown in a study that water storage and thermocycle did not affect the bond strength of GIC to Er-YAG prepared dentin, while a reduction was observed on bur-cut dentin [208]. Pretreatment with dentin conditioner can be advantageous to increase the retention of GIC to laser-irradiated dentin [209].

The majority of bond strength studies are done on flat dentin surfaces, which is an ideal condition compared to clinical situations. However, light-cured RBCs produce polymerization stress in high C-factor cavities, leading to separation from the cavity wall [210]. Yao et al. [211,212] compared the performance of self-adhesive restorative materials and RMGICs that were applied to flat dentin and a Class I cavity with a high C-factor. Lower bond strengths were reported when applied to the cavity, along with high pretest failures. Moreover, self-curing had more favorable results than light-curing, most likely related to shrinkage stress [211]. Further studies with simulation of clinical applications are necessary. 

Quantification of the bond strength is mainly divided into macro and micro designs, depending on the size of the bonded area. Shear bond strength (SBS) and microtensile bond strength (μTBS) tests were the commonly preferred testing methods for the evaluation of the adhesion of GICs. Bond strength values depend on the test method used; smaller cross-sectional areas used in micro-tests were associated with higher bond strengths [213] due to the reduced flaws and defects within the smaller interface. These defects may act as stress points that initiate the cracks [196]. Although the μTBS test is a commonly used laboratory test, its reliability should be discussed when GICs are being tested. Cutting and trimming procedures during the specimen preparation for μTBS may cause damage to the brittle materials. The advantages of the shear bond strength test should be considered when testing brittle materials such as GICs. In addition, it is essential not to directly compare the results of testing methods with different cross-sectional areas without involving sound fracture mechanics and Weibull analysis [214]. 

The mechanical tests assessing the bond strength of GICs tend to show cohesive failure within the material [92,93,137,215]. Cohesive failures indicate that the bond strength represents the strength of the material rather than the interface. A fine layer of GIC was observed on the dentin surface of the cohesively failed specimens [216], which was related to the ion-enriched interaction zone [217]. Therefore, failure mode analysis is very critical for the evaluation of GICs and RMGICs. The dominant failure type, together with the comprehensive analysis, should be reported in studies. 

Bond strength tests were performed most commonly at 24 h in the evaluated studies. However, GICs might not reach their optimum strength in such a short time. In our opinion, water storage for 1 week [79,90,96,135,158,159,167,169,172,173,203,207,211,212,218] rather than 24 h for the evaluation of short-term performance is important considering the maturation of GICs. On the other hand, for a better prediction of the clinical performance, aged bond strength should be measured rather than the immediate bond strength [219]. Due to the long-term maturation of GICs, water or artificial saliva storage might be a better aging method compared to accelerated aging methods. 

## 4. Conclusions

It is challenging for clinicians to make choices among the wide variety of material types and brands. The decision-making process should include a comprehensive evaluation of the patient-related factors and material knowledge to ensure durable restorations. Although RBCs perform better, GICs and RMGICs can be considered as a good alternative, especially in pediatric and geriatric dentistry, when there is demand for a faster and less technique-sensitive procedure. Different test protocols for the evaluation of the bonding performance to altered dentin tissues make it difficult to obtain a fair comparison of the materials in vitro. It is also not possible to make a direct assumption according to the limited number of studies on the effect of contamination on bond strength of GICs and RMGICs; however, reconditioning with PAA may be recommended as a remedy for contamination. There is still need for long-term evaluation of GICs with standard protocols considering their maturation over time. 

## Figures and Tables

**Table 1:** Features of the laboratory models for testing caries lesions.

**NCAD**			Clinically relevant Structural variability—standardization is challenging	
**ACAD**	**Chemical**	**pH cycling**	Demineralization–remineralization cycles to mimic oral environment The duration of pH cycling ranges from a few days to weeks	Simple, fast and low-cost Homogeneous and controlled Unable to completely simulate the complex oral conditions Useful for material evaluation and different variables Mostly commonly lactic or acetic acid gel or solution
		**Static**	Incubation into an acidified solution or gel under static conditions for a period	
	**Microbiological**		Involve bacterial biofilms formation Most commonly used bacteria is *Streptococcus mutans* Hard to standardize Unable to completely simulate the complex oral conditions	

NCAD: natural caries-affected dentin, ACAD: artificial caries-affected dentin.

**Table 2:** Summary of the included studies comparing the bond strength of sound and CAD/demineralized dentin.

**First Author, Year**	**Tooth Type**	**Substrate**	**Test Method**	**Storage**	**Material**	**Significance**	
Khor, 2022 [137]	permanent	ACAD (pH cycling)	μTBS	48 h	GIC (Fuji IX GP Extra)	SD > CAD	
Al-Hasan, 2022 [138]	permanent	NCAD	SBS	24 h or 2 months	RMGIC (Fuji II LC) RMGIC (Activa— without adhesive)	24 h: SD > CAD SD = CAD	2 m: CAD > SD SD = CAD
Al-Taee, 2022 [131]	permanent	NCAD	SBS	24 h or 3 months	GIC (Fuji IX GP) RMGIC (Fuji II LC) RMGIC (experimental) RBC (Filtek Supreme + Scothbond Universal)	24 h: SD < CAD SD < CAD SD < CAD SD < CAD	3 m: SD = CAD SD = CAD SD > CAD SD > CAD
Keskin, 2021 [91]	permanent	ACAD (pH cycling)	μTBS	24 h + 10,000 TC	GIC (Fuji IX Extra) GIC (Equia Forte) RMGIC (Fuji II LC) RMGIC (Activa) Giomer (Beautifill II LS)	SD > CAD SD = CAD SD > CAD SD > CAD SD = CAD	
Jiang, 2020 [135]	permanent	ACAD (microbiological)	μTBS	7 days	GIC (Ketac Molar)	SD > CAD	
Ng, 2020 [139]	permanent	ACAD (static)	SBS	24 h	GIC (Fuji IX)	SD = CAD	
Ahangari, 2020 [134]	permanent	ACAD (static)	μTBS	N/A	GIC (Equia Forte) GIC (Ketac Molar) RMGIC (capsulated Fuji II LC) RMGIC (hand-mixed Fuji II LC) Flowable RBC (Filtek Z350)	SD > CAD SD > CAD SD > CAD SD > CAD SD > CAD	
Tedesco, 2018 [83]	deciduous	Erosion	μSBS	24 h or 12 months	GIC (Fuji IX) RMGIC (Vitremer) RBC (Filtek Z250 + Adper Single Bond 2)	24 h: SD = eroded SD > eroded SD > eroded	12 m: SD = eroded SD > eroded SD > eroded
El-Deeb, 2018 [129]	permanent	NCAD	μSBS	24 h	GIC (GC Fuji IX GP Fast) GIC (Fuji IX GP containing CHX) GIC (ChemFil Rock)	SD = CAD SD = CAD SD = CAD	
Agob, 2018 [140]	permanent	ACAD (static)	μSBS	24 h	RMGIC (Fuji II LC)	SD > CAD	
Saad, 2017 [133]	permanent	ACAD (static)	μTBS	24 h or 3 months	RMGIC (Fuji II LC) No conditioner Conditioner	24 h: SD > CAD SD > CAD	3 m: SD = CAD SD > CAD
Kucukyilmaz, 2017 [141]	deciduous	ACAD (pH cycling)	μTBS	24 h	GIC (Equia) GIC (GCP Glass Fill) RMGIC (Ketac N100)	SD > CAD SD = CAD SD > CAD	
Hamama, 2015 [130]	permanent	NCAD (prepared with rotary instruments)	μTBS	24 h	RMGIC (Riva LC RMGIC + Riva Bond LC) RMGIC (Fuji II LC + Fuji Bond LC)	SD = CAD SD = CAD	
Aykut-Yetkiner, 2015 [132]	permanent	NCAD	μTBS	24 h	GIC (Ketac Molar) RMGIC (Vitremer) Bioactive restorative material (Surefil (with adhesive))	SD = CAD SD = CAD SD = CAD	
Calvo, 2014 [97]	deciduous	ACAD (pH cycling)	μTBS	24 h or 2 years	GIC (Ketac Molar Easy Mix) RMGIC (Vitremer) RMGIC (Ketac Nano)	24 h: SD = CAD SD = CAD SD = CAD	2 y: SD = CAD SD = CAD SD = CAD
Alves, 2013 [98]	deciduous	ACAD (pH cycling)	μTBS	24 h	GIC (Ketac Molar Easy Mix) RMGIC (Ketac N100) RMGIC (Vitremer)	SD = CAD SD = CAD SD = CAD	
Lenzi, 2013 [136]	deciduous	ACAD (pH cycling)	μTBS	24 h	GIC (Fuji IX) 1 layer 2 layers	SD = CAD SD > CAD	
Cruz, 2012 [142]	permanent	Erosion	μSBS	24 h	GIC (Ketac Molar Easy Mix) RMGIC (Vitremer) RBC (Adper Single Bond 2 + Filtek Z250)	SD = eroded SD = eroded SD = eroded	
Marquezan, 2010 [85]	deciduous	ACAD (pH cycling)	μTBS	24 h or load cycling or pH cycling	RMGIC (Vitremer) RBC (Adper Single Bond 2 + Filtek Z100)	All storage times: SD = CAD SD > CAD	
Czarnecka, 2007 [143]	permanent	NCAD (sclerotic dentin)	SBS	24 h	GIC (Fuji IX GP) GIC (Fuji IX capsulated) GIC (Fuji IX Fast capsulated) GIC (Ketac Molar) GIC (Ketac Molar Aplicap)	SD > CAD SD = CAD SD = CAD SD > CAD SD = CAD	
Choi, 2006 [93]	permanent	ACAD (static)	μTBS	24 h	GIC (Ketac-Fil Plus Aplicap) RMGIC (Photac-Fil Aplicap)	SD > CAD SD > CAD	
Cehreli, 2003 [87]	deciduous	ACAD (static)	μTBS	18 months	RMGIC (Vitremer) PMRC (Dyract AP) PMRC (Compoglass F) PMRC (F2000)	SD > CAD SD > CAD SD > CAD SD > CAD	
Burrow, 2003 [94]	permanent	NCAD (prepared with chemomechanical caries removal)	μTBS	24 h	GIC (Fuji IX) RMGIC (Fuji II LC) RBC (Clearfil SE Bond + RBC) RBC (One Coat Bond + RBC)	SD = CAD SD > CAD SD = CAD SD = CAD	

SD: sound dentin, CAD: caries-affected dentin, NCAD: natural caries-affected dentin, ACAD: artificial caries-affected dentin, GIC: glass ionomer cement, RMGIC: resin-modified glass ionomer cement, RBC: resin-based composite, PMRC: poliasid-modified resin composite, μTBS: microtensile bond strength, SBS: shear bond strength, μSBS: microshear bond strength.

**Table 3:** Summary of the included studies evaluating the effect of contamination on bond strength of direct restorative materials.

**First Author, Year**	**Material**	**Contamination Protocol**	**Storage**	**Test Method**	**Main Findings**
Almeida, 2021 [154]	RMGIC (Riva Light Cure)	Contaminant: Saliva, blood, hemostatic agent Actively applied by brush for 2 min For decontamination, rinsing and drying for 20 s or reconditioning with PAA for 10 s.	7 days or 10,000 TC	μTBS	Contaminants impaired the bond strength. Reconditioning was effective for decontaminating saliva and hemostatic agent, but not for blood.
Shimazu, 2020 [156]	RBC (Clearfil AP-X + OptiBond Solo Plus) RBC (Clearfil AP-X +Scotchbond Universal) RMGIC (Fuji II LC) GIC (Fuji IX extra)	Contaminant: Artificial saliva Mild—0.1 mL of saliva was placed and dried slightly Severe—0.1 mL of the saliva was used as is.	24 h	μTBS	No effect on GIC and RMGIC, reduced bond strength of RBC.
Saad, 2019 [161]	RBC (Clearfil AP-X + Clearfil SE Bond) RMGIC (Fuji II LC)	Contaminant: Hemostatic agents applied for 5 min, rinsed with water for 20 s. Application of different conditioners: RBC—Clearfil SE bond RMGIC—Cavity Conditioner, Self Conditioner or Clearfil SE Primer	24 h	μTBS	No effect of contaminant on RMGIC.
Shimazu, 2014 [155]	RBC (Clearfil AP-X + OptiBond Solo Plus) RBC (Clearfil AP-X + Clearfil S3) RMGIC (Fuji II LC) GIC (Fuji IX ekstra)	Contaminant: Artificial saliva Mild—0.1 mL of saliva was placed and dried slightly Severe—0.1 mL of the saliva was used as is.	24 h	SBS	No effect on GIC and RMGIC, reduced bond strength of RBC.
Dursun, 2011 [157]	RMGIC (Fuji II LC + i Bond)	Contaminant: Water, saliva Water or saliva was applied with brush before or after adhesive application and air-thinned, leaving the specimen visibly moist.	24 h	SBS	No effect.
Wangpermtam, 2011 [162]	RMGIC (Fuji II LC)	Contaminant: Provisional cement, handpiece lubricant Provisional cement applied with hand instrument, removed 3 min after setting with the same instrument, or two drops of lubricant covering the surface, wiped off with sponge. For decontamination, Hibiscrub, CHX or pumice were used. A drop of Hibiscrub or CHX applied with a brush for 1 min, washed and conditioned with 10% PAA. Pumice paste was rubbed with brush for 1 min, conditioned with 10% PAA.	24 h	μTBS	Provisional cement and handpiece lubricant reduced the bond strength. Hibiscrub was more effective for decontaminating the lubricant.
Kulczyk, 2005 [158]	GIC (Fuji IX GP) GIC (Ketac Molar)	Contaminant: Saliva An amount of 0.05 mL of saliva was applied for 20 s. For decontamination, surface was washed with water for 5 s and gently air-dried.	7 days	SBS	No effect.
Safar, 1999 [159]	RMGIC (Fuji II LC)	Contaminant: Saliva Saliva was applied on surface for 10 s and thinned with air until only a thin, wet film remained. For decontamination, it was either dried, rinsed or reconditioned.	7 days	SBS	Saliva contamination reduced the bond strength. Decontamination methods failed to recover it.
Aboush, 1987 [160]	GIC (ChemFil II)	Contaminant: Saliva Immersed in saliva for 1 min or 10 days, then washed for 20 s with water and air-dried. For decontamination, 25% PAA for 30 s, 50% citric acid solution for 30 s or pumice applied for 10 s using a rubber cup. All specimens were then rinsed off and air-dried.	24 h	TBS	Saliva contamination was detrimental to bond strength. PAA, citric acid and pumice were similarly effective for decontamination.

## Data Availability

Data sharing is not applicable.
